# Altered host immunity, human T lymphotropic virus type I replication, and risk of adult T-cell leukemia/lymphoma: a prospective analysis from the ATL Cohort Consortium

**DOI:** 10.1186/1742-4690-8-S1-A81

**Published:** 2011-06-06

**Authors:** Brenda M Birmann, Akihiko Okayama, Norma Kim, Kokichi Arisawa, Elizabeth C Breen, Anna B F Carneiro-Proietti, Kerstin I Falk, Barrie Hanchard, Manami Inoue, Otoniel Martínez-Maza, Edward L Murphy, Ruth M Pfeiffer, Takashi Sawada, Sherri O Stuver, Shoichiro Tsugane, Hongchuan Li, Catherine A Suppan, Nancy E Mueller, Michie Hisada

**Affiliations:** 1 Channing Laboratory, Department of Medicine, Brigham and Women’s Hospital and Harvard Medical School, Boston, Massachusetts, 02115, USA; 2 Department of Rheumatology, Infectious Diseases and Laboratory Medicine, University of Miyazaki, Miyazaki, Japan; 3 RTI International, Rockville, Maryland, 20852, USA; 4 Department of Preventive Medicine, Institute of Health Biosciences, Tokushima University, Tokushima, Japan; 5 Cousins Center for Psychoneuroimmunology, Department of Psychiatry and Biobehavioral Sciences, David Geffen School of Medicine at UCLA, University of California Los Angeles, Los Angeles, California, 90095, USA; 6 Hemominas Foundation, Belo Horizonte, Minas Gerais, Brazil; 7 Department of Preparedness, Swedish Institute for Communicable Disease Control and MTC, Karolinska Institute, Stockholm, Sweden; 8 Department of Pathology, University of the West Indies, Mona Kingston, Jamaica; 9 Research Center for Cancer Prevention and Screening, National Cancer Center, Japan; 10 Departments of Obstetrics and Gynecology, and Microbiology, Immunology and Molecular Genetics, David Geffen School of Medicine at UCLA, and Department of Epidemiology, UCLA School of Public Health, University of California Los Angeles, Los Angeles, California, 90095, USA; 11 Departments of Laboratory Medicine and Epidemiology/Biostatistics, University of California San Francisco and Blood Systems Research Institute, San Francisco, California, 94118, USA; 12 Division of Cancer Epidemiology and Genetics, National Cancer Institute, NIH, DHHS, Bethesda, Maryland, 20892, USA; 13 Department of Clinical Development, Oncology Product Creation Unit, Eisai Co. Ltd., Tokyo, Japan; 14 Department of Epidemiology, Boston University School of Public Health, Boston, Massachusetts, 02118, USA; 15 Laboratory of Experimental Immunology, Cancer and Inflammation Program, Center for Cancer Research, National Cancer Institute, NIH, DHHS, Frederick, MD, 21702, USA; 16 Basic Research Program, SAIC-Frederick Inc., National Cancer Institute-Frederick, Frederick MD, 21702, USA; 17 Department of Epidemiology, Harvard School of Public Health, Boston, Massachusetts, 02115, USA; 18 Current affiliation: Takeda Global Research and Development Center, Inc., Deerfield, Illinois, 60015, USA

## Background

Adult T-cell leukemia/lymphoma (ATL) is a rare and often fatal outcome of infection with human T-lymphotropic virus type I (HTLV-I). Altered host immunity in HTLV-I carriers has been postulated as a risk factor for ATL, but is not well understood.

## Methods

We prospectively examined well-validated serologic markers of HTLV-I pathogenesis and host immunity in 53 incident ATL cases and 150 carefully matched asymptomatic HTLV-I carriers from eight population-based studies in Japan, Jamaica, the United States and Brazil. We used multivariable conditional logistic regression, conditioned on the matching factors (cohort/race, age, sex, and sample collection year), to evaluate the biomarkers’ associations with ATL in all subjects and by years (≤5, >5) from blood draw to ATL diagnosis.

## Results

In the pooled population, above-median soluble interleukin-2-receptor-alpha levels (sIL2R, v. ≤ median; odds ratio (OR), 95% confidence interval (CI)=4.08, 1.47-11.29) and anti-Tax seropositivity (anti-Tax; OR, 95% CI=2.97, 1.15-7.67), which indicate T cell activation and HTLV-I replication, respectively, were independently associated with an increased ATL risk. Above-median total immunoglobulin E levels (v. ≤ median; OR, 95% CI=0.45, 0.19-1.06), which indicate type 2 (B cell) activation, predicted a lower ATL risk. The sIL2R and anti-Tax associations with ATL were stronger in samples collected ≤5 years pre-diagnosis.

## Conclusions

The biomarker profile predictive of ATL risk suggests a role for heightened T cell activation and HTLV-I replication and diminished type 2 immunity in the etiology of ATL in HTLV-I carriers. Translation of these findings to clinical risk prediction or early ATL detection requires further investigation.

